# The Role of Heart Rate on the Associations Between Body Composition and Heart Rate Variability in Children With Overweight/Obesity: The ActiveBrains Project

**DOI:** 10.3389/fphys.2019.00895

**Published:** 2019-07-16

**Authors:** Abel Plaza-Florido, Jairo H. Migueles, Jose Mora-Gonzalez, Pablo Molina-Garcia, Maria Rodriguez-Ayllon, Cristina Cadenas-Sanchez, Irene Esteban-Cornejo, Socorro Navarrete, Rosa Maria Lozano, Nathalie Michels, Jerzy Sacha, Francisco B. Ortega

**Affiliations:** ^1^PROFITH “PROmoting FITness and Health Through Physical Activity” Research Group, Sport and Health University Research Institute (iMUDS), Department of Physical and Sports Education, Faculty of Sport Sciences, University of Granada, Granada, Spain; ^2^Department of Rehabilitation Sciences, KU Leuven, Leuven, Belgium; ^3^Center for Cognitive and Brain Health, Department of Psychology, Northeastern University, Boston, MA, United States; ^4^Andalusian Centre of Sport Medicine (CAMD), Junta de Andalucía, Granada, Spain; ^5^Department of Public Health, Faculty of Medicine and Health Sciences, Ghent University, Ghent, Belgium; ^6^Faculty of Physical Education and Physiotherapy, Opole University of Technology, Opole, Poland; ^7^Department of Cardiology, University Hospital, University of Opole, Opole, Poland; ^8^Department of Biosciences and Nutrition, Karolinska Institutet, Huddinge, Sweden

**Keywords:** parasympathetic, sympathetic, fat mass, adiposity, preadolescents

## Abstract

**Background:**

Heart rate variability (HRV) is negatively associated with body mass index and adiposity in several populations. However, less information is available about this association in children with overweight and obesity, especially severe/morbid obesity, taking into consideration the dependence of HRV on heart rate (HR).

**Objectives:**

(1) to examine associations between body composition measures and HRV, (2) to study differences in HRV between children with overweight and severe/morbid obesity; and (3) to test whether relationships and differences tested in objectives 1 and 2, respectively are explained by the dependency of HRV on HR.

**Methods:**

A total of 107 children with overweight/obesity (58% boys, 10.03 ± 1.13 years) participated in this study. Body composition measures were evaluated by Dual-energy X-ray absorptiometry (DXA). HRV parameters were measured with Polar RS800CX^®^.

**Results:**

Body composition measures were negatively associated with HRV indicators of parasympathetic activity (β values ranging from −0.207 to −0.307, all *p* < 0.05). Children with severe/morbid obesity presented lower HRV values with respect to children with overweight/mild obesity in HRV parameters indicators of parasympathetic activity (*p* = 0.035). All associations disappeared after further adjustment for HR (all *p* > 0.05).

**Conclusion:**

All associations between adiposity/obesity and HRV could be explained by HR, suggesting a key confounding role of HR in HRV studies in children with weight disturbances.

## Introduction

Childhood obesity is one of the major public health concerns worldwide (World Health Organization [WHO], 2017). Evidence points out that pediatric obesity, especially severe obesity, is a risk factor for the development of insulin resistance, low high-density lipoprotein cholesterol levels and high systolic blood pressure, among others (Wells et al., 2004; Skinner et al., 2015). Low parasympathetic activity (PA) has been also proposed as an important risk factor for cardiovascular disease and mortality (Thayer and Lane, 2007; Thayer et al., 2010) and related to several body composition (BC) measures (Gutin et al., 2000; Nagai et al., 2003; Rodríguez-Colón et al., 2011). PA can be assessed by measuring heart rate variability (HRV) which refers to the variability in the time interval between consecutive heart beats (variability of the R−R interval) (Task Force, 1996), so that lower HRV values indicate a lower PA.

A negative association has been reported between several BC measures [i.e., fat mass and fat-free mass (Gutin et al., 2000), body weight, and waist circumference (WC) (Rodríguez-Colón et al., 2011), and body mass index (BMI) (Birch et al., 2012)] with HRV parameters in time- and frequency-domain (hereinafter referred to as HRV parameters) either in children with normal weight (NW), overweight (OW), or obesity (OB). Accordingly, some studies found lower HRV values in children with OB compared to their peers with NW and OW (Martini et al., 2001; Nagai et al., 2003; Rabbia et al., 2003; Kaufman et al., 2007; Rodríguez-Colón et al., 2011). Conversely, Redón et al. (2016) did not find significant differences in HRV across different weight status groups (i.e., children with OW, mild OB and severe OB). These controversies can be explained by methodological differences between studies (i.e., time of recording HRV, the instrument employed to record HRV, children with different weight status). Otherwise, to the best of our knowledge, the inclusion of subgroups of children with severe/morbid OB is currently lacking with only one study including children with severe obesity (Redón et al., 2016). Importantly, HRV strongly depends on heart rate (HR) (Sacha and Pluta, 2008; Sacha, 2013, 2014b). This dependency has been shown to be an important factor to consider when analyzing HRV associations with health outcomes (Trimmel et al., 2015), but previous studies have failed in considering this dependency. This is particularly important in this population because those often have higher HR, and thus there is a higher chance of HRV underestimation.

Therefore, the aims of this study were: (1) to examine associations between BC measures (i.e., BMI, WC, WC/height, fat mass index, body fat percentage, fat free mass index, and visceral adipose tissue) and HRV parameters in time and frequency-domain, (2) to study differences in HRV across children with different weight status, and (3) to test whether the relationships between BC measures and HRV are explained by the dependency of HRV on HR in children with OW/OB.

## Materials and Methods

### Study Design and Participants

This cross-sectional study used baseline data of 107 children with OW/OB (9–11 years, 58% boys) from the ActiveBrains project^1^ (Cadenas-Sánchez et al., 2016). The age group (9–11 years) was made on a preadolescent sample due to adolescent physiological and psychological changes are dramatic, and it is difficult to control confounding factors. Detailed design and methods have been described elsewhere (Cadenas-Sánchez et al., 2016). For feasibility reasons, the ActiveBrains project was conducted in three waves, carrying out the baseline assessments from October 2014 to February 2016. This study was conducted according to the Declaration of Helsinki. The protocol was approved by the Committee for Research Involving Human Subjects at the University of Granada (Reference: 848, February 2014). All parents had received information about the study and gave written informed consent in accordance with the Declaration of Helsinki.

### Body Composition Measures

Body weight and height were measured with an electronic scale and a stadiometer (Seca instruments, Germany, Ltd.). BMI was calculated as kg/m^2^ and participants were then classified as children with OW, mild, severe or morbid OB according to the sex- and age-specific international BMI standards (World Obesity Federation) (Cole and Lobstein, 2012; Bervoets and Massa, 2014). We combined children with severe and morbid OB due to their low prevalence in our sample (i.e., severe OB: *n* = 22, 20.6%; morbid OB: *n* = 11, 10.3%). WC was evaluated as an indicator of central fat using the International Society for the Advancement of Kinanthropometry (ISAK) procedures (ISAK, 2006). Body weight, height, and WC were collected twice consecutively by the same trained evaluator, and the average for each parameter was used. Fat mas and fat-free mass were measured by dual energy X-ray absorptiometry (DXA, Discovery densitometer from Hologic). Fat mass index, fat-free mass index and body fat percentage (FMI, FFMI, and BFP, respectively) were derived as the ratio between fat mass and fat-free mass (kg) with the squared height (m^2^), and the percentage (%) of adipose tissue relative to body weight, respectively. Also, visceral adipose tissue (VAT) was measured with DXA.

### Heart Rate Variability

Participants were placed supine for 10 min in a quiet and comfortable room between 9 a.m. and 12 p.m. Supine position has shown a higher reliability for the HRV measurement in children than sitting or standing (Silva et al., 2016). The POLAR RS800CX (Polar Electro Oy Inc., Kempele, Finland) recorded HRV during 10 min at a sampling frequency of 1000 Hz. This HR monitor provides valid measures with respect to ECG (Gamelin et al., 2008) and it is reliable for the HRV assessment in children and adolescents (Vasconcellos et al., 2015). Participants were encouraged to breathe normal, keep relaxed and to not move or speak during the evaluation.

We used the normal R–R intervals after excluding the extreme values with the automatized low filter available in the Kubios software (HRV analysis, University of Eastern Finland) (Niskanen et al., 2004; Tarvainen et al., 2014). The R–R interval series were detrended using the smoothness prior method with alpha set at 500 and a cubic interpolation at the default rate of 4 Hz. Out of the 10 min recorded, the middle 5 min (i.e., from minutes 3 to 8) were checked for quality (i.e., normal distribution of the R–R intervals, no large R–R interval outliers and R–R intervals equidistance and minimal variation) and a different period of 5-min was selected when necessary. We derived time- and frequency-domain HRV parameters based on the Guidelines of Task Force of The European Society of Cardiology and The North American Society of Pacing and Electrophysiology (Task Force, 1996). In the time-domain, we computed the squared root of the mean of the sum of the squares of successive normal R–R interval differences (RMSSD) and the percentage number of pairs of adjacent normal R–R intervals differing by more than 50 ms in the entire recording (pNN50) as indexes of PA. We also computed the standard deviation of all normal R–R intervals (SDNN). In the frequency-domain, we performed spectral analyses using the non-parametric fast Fourier transformation algorithm (FFT), with Welch’s periodogram method (i.e., 50% overlap Hanning window as pre-processing technique and calculating area under the curve with an integration). We derived total power (TP) of HRV spectrum, the power in the high (HF) and the low frequency (LF) bands (TP: 0–0.4 Hz; LF: 0.04–0.15 Hz; HF: 0.15–0.4 Hz) in absolute units (ms^2^). HF and the LF/HF ratio were then used in the analyses as indicators of PA and sympatho-vagal balance, respectively (Task Force, 1996), although physiological significance of LF/HF ratio is not clear (Goldstein et al., 2011; Billman, 2013).

To remove the HRV dependence on HR, we calculated the corrected HRV parameters proposed by Sacha et al. (2013) based on two assumptions: (1) If HRV parameters were negatively correlated with HR, the correction procedure consisted in calculating ratios between HRV parameters and different powers of their corresponding mean normal R–R interval; (2) if HRV parameters were positively correlated with HR, the correction procedure was performed by multiplying HRV parameters by the adequate powers of mean normal R–R interval as follows: RMMSDc = RMSSD/meanRR^3.7^, pNN50c = pNN50/meanRR^5.3^, SDNNc = SDNN/meanRR^2.8^, FFT TPc = TP/meanRR^6.3^, FFT HFc = HF/meanRR^6.3^, FFT LFc = LF/meanRR^5.0^, FFT LF/HFc = LF/HF x meanRR^1.8^.

### Basic Confounders

Peak height velocity (PHV) was derived from measured height and seated height as a discriminant measure of maturational status (Malina et al., 2015; Moore et al., 2015). Maturity offset was calculated by subtracting the PHV age from the chronological age and used in the analyses.

Socioeconomic status was assessed by parental education level. Both mother and father self-reported their highest educational level as no elementary school, elementary school, middle school, high school, and university degree completed. Parent responses were then combined as follows: none of the parents had a university degree, one of the parents had a university degree or both parents had a university degree.

Since each wave of participants was evaluated in different months of the year, and it could affect to HRV, we decided to add wave as a dummy covariate in our models to control for the potential seasonal variance in the outcomes.

### Statistical Analyses

Descriptive characteristics are presented as means and standard deviations (mean ± SD) for continuous variables that exhibited a normal distribution, medians, and interquartile ranges for continuous non-normal variables, and frequencies, and percentages for categorical variables. Values of corrected HRV parameters were presented as medians with the 5th and 95th percentiles in the same way that were presented previously by Ga̧sior et al. (2018) in healthy weight children. Kolmogorov–Smirnov test and a visual inspection of histograms were performed to assess the normal distribution of variables. Normal scores of HRV parameters were calculated according to the Blom formula (Blom, 1954, 1958) to obtain normally distributed variables when needed.

Spearman correlation tests were performed to study associations between standard (without correction by HR) and corrected HRV parameters with mean HR to confirm their dependence and their loss of dependency after performing the Sacha et al. (2013) correction. Sex, PHV offset, parent education university level, and wave (transformed into binary variables) were selected as basic confounders. Stepwise linear regression models were performed to test the relevance of basic confounders in all models, only variables included in the stepwise models were carried forward to the final model with BC measures as predictors and HRV parameters as outcomes. Associations of BC measures with HRV parameters were studied using linear regressions. Model 1 included only the selected basic confounders by the stepwise models, and standard HRV parameters as outcomes. Model 2 included the same confounders than model 1 plus mean HR, and standard HRV as outcomes (Van De Wielle and Michels, 2017). Lastly, model 3 included the selected basic confounders by the stepwise models, and corrected HRV parameters as outcomes (Sacha et al., 2013).

Finally, we used analysis of covariance (ANCOVA) to explore differences in HRV parameters across weight status groups (i.e., OW, mild, severe/morbid OB). ANCOVA models were adjusted for the same confounders introduced in linear regression models 1 and 2. We only performed ANCOVA adjusted by HR (model 2) and did not show this analysis with corrected HRV parameters (HRV_c_) (model 3) as the results were the same independently of the method performed to remove the dependence of HRV on HR. Statistical significance was defined at the level of *p* < 0.05 for all the analyses. Analyses were performed using SPSS version 21.0 (IBM Corporation, NY, United States).

## Results

Table 1 shows the descriptive characteristics of participants stratified by weight status (i.e., OW, mild, severe/morbid OB groups). As expected, BC measures showed significant differences between weight status groups (all *p* ≤ 0.001). Also, children with severe/morbid OB presented significantly higher mean HR than their peers with OW and mild OB (*p* < 0.002 and *p* < 0.001, respectively). For descriptive purposes, Table 2 compares corrected HRV parameters analyzed in our study with normative corrected HRV parameters recently published in healthy weight children (Ga̧sior et al., 2018). It can be observed that our sample of children with OW/OB have lower values of HRV than healthy weight children.

**TABLE 1:** Descriptive characteristics of participants.

**Variables**	**Total sample (*n* = 107)**	**Overweight (*n* = 28, 26%)**	**Mild-obesity (*n* = 46, 43%)**	**Severe/Morbid-obesity (*n* = 33, 31%)**	***P* value**
Boys, *n* (%)	62 (58)	16 (57)	29 (63)	17 (52)	0.592
**Parents with university degree, *n* (%)**					0.005^∗∗^
None of them	71 (66)	15 (54)	27 (59)	29 (88)^a,b^	
One of them	19 (18)	6 (21)	10 (22)	3 (9)^a,b^	
Both of them	17 (16)	7 (25)	9 (20)	1 (3)^a,b^	
**Wave, *n* (%)**					0.905
Wave 1	19 (18)	5 (18)	8 (17)	6 (18)	
Wave 2	43 (40)	10 (36)	20 (44)	13 (39)	
Wave 3	45 (42)	13 (46)	18 (39)	14 (42)	
Age (years)	10 ± 1	10 ± 1	10 ± 1	9 ± 1^a,b^	0.004^∗∗^
PHV offset (years)	−2.26 ± 0.99	−2.25 ± 1.01	−2.06 ± 1.02	−2.54 ± 0.90	0.107
**Body composition**					
Weight (kg)	56 ± 11	46 ± 7	58 ± 10^a^	62 ± 9^a^	< 0.001^**^
Height (cm)	144 ± 8	142 ± 9	147 ± 8^a^	142 ± 7^b^	0.014^∗∗^
BMI (kg/m^2^)	27 ± 3	22 ± 1	26 ± 2^a^	31 ± 2^a,b^	< 0.001^**^
WC (cm)	90 ± 10	80 ± 6	90 ± 8^a^	98 ± 7^a,b^	< 0.001^**^
WC/Height	0.62 ± 0.06	0.57 ± 0.03	0.62 ± 0.04^a^	0.69 ± 0.04^a,b^	< 0.001^**^
DXA FM (Kg)	25 ± 7	18 ± 4	25 ± 5^a^	30 ± 7^a,b^	< 0.001^**^
DXA FMI (kg/m^2^)	12 ± 3	9 ± 1	11 ± 2^a^	15 ± 2^a,b^	< 0.001^**^
DXA BFP (%)	44 ± 6	39 ± 4	43 ± 4^a^	49 ± 4^a,b^	< 0.001^**^
DXA FFM (Kg)	31 ± 5	28 ± 5	32 ± 6^a^	31 ± 4^a^	< 0.001^**^
DXA FFMI (kg/m^2^)	15 ± 1	14 ± 1	15 ± 1^a^	15 ± 1^a^	< 0.001^**^
DXA Total VAT (g)^*^	398 ± 115	297 ± 73	397 ± 97^a^	484 ± 99^a,b^	< 0.001^**^
DXA VAT FMI (g)^*^	191 ± 51	146 ± 33	182 ± 34^a^	240 ± 41^a,b^	< 0.001^**^
**Heart rate variability**					
Mean HR (bpm)	81 ± 10	79 ± 10	79 ± 9	87 ± 8^a,b^	< 0.001^**^
Mean RR (ms)	747 ± 91	774 ± 101	767 ± 87	695 ± 63^a,b^	< 0.001^**^
RMSSD (ms)	60 [57]	71 [55]	72 [83]	51 [28]	0.056
pNN50 (%)	31 [38]	41 [38]	37 [43]	21 [19]	0.023^∗∗^
SDNN (ms)	60 [42]	68 [44]	65 [54]	54 [25]	0.299
FFT TP (ms^2^)	3068 [5405]	3736 [5609]	4084 [5997]	2349 [2339]^a^	0.035^∗∗^
FFT HF (ms^2^)	1223 [2642]	1987 [3212]	2140 [3731]	860 [814]	0.380
FFT LF (ms^2^)	1288 [1724]	1291 [2023]	1409 [1903]	1151 [1195]	0.577
FFT LF/HF	0.9 [1.2]	0.8 [1.2]	0.8 [1.0]	1.5 [1.5]	0.061

Data presented as mean ± SD, as number and frequency. Median [IQR: Interquartile range] are presented for standard HRV parameters because these variables presented skewed distributions. PHV, peak height velocity; BMI, body mass index; WC, waist circumference; DXA, dual energy X-ray absorptiometry; FM: fat mass; FMI, fat mass index; BFP, body fat percentage; FFM, fat-free mass; FFMI, fat-free mass index; VAT, visceral adipose tissue; HR, heart rate; RR, R–R intervals; RMSSD, The square root of the mean of the sum of the squares of differences between adjacent R–R intervals; pNN50: percentage (%) of the total pairs of R–R intervals that differ by more than 50 ms; SDNN, standard deviation of all normal R–R intervals; FFT, fast fourier transformation; HF, high frequency band (0.15–0.4 Hz) index of parasympathetic activity; LF, low frequency band (0.04–0.15 Hz) mix index of sympathetic and parasympathetic activity. ^*^Indicates that these variables have some missing values, i.e., DXA Total VAT (*g*): *n* = 19; DXA VAT FMI (*g*): *n* = 19; *P* value column denotes significant differences among groups. ^*a*^Denotes significant differences (*p* < 0.05) respect to the subgroup of children with overweight. ^*b*^Denotes significant differences (*p* < 0.05) respect to the subgroup of children with mild obesity. ^∗∗^*p* < 0.05.

**TABLE 2:** Normative values of corrected HRV parameters in children with overweight/obesity and values published previously in healthy weight children (Ga̧sior et al., 2018).

**Overweight/obesity (*n* = 107)**				**Healthy children (*n* = 312)**		
**HRV PARAMETER**	**Median**	**5th percentile**	**95th percentile**	**Median**	**5th percentile**	**95th percentile**
RMSSD_c_	1.4E-09	7.1E-10	2.6E-09	1.4E-07	7.6E-08	2.6E-07
pNN50_c_	1.6E-14	2.0E-15	3.6E-14	1.4E-13	2.0E-14	3.0E-13
SDNN_c_	5.6E-07	2.6E-07	8.7E-07	2.6E-05	1.5E-05	4.6E-05
FFT TP_c_	3.0E-15	1.0E-15	7.0E-15	1.2E-11	3.6E-12	3.8E-11
FFT HF_c_	1.1E-15	3.0E-16	3.3E-15	6.8E-12	1.7E-12	2.5E-11
FFT LF_c_	5.2E-12	1.1E-12	1.8E-11	2.9E-09	7.5E-10	9.8E-09
FFT LF/HF	1.4E+05	3.5E+04	4.4E+05	4.5E+02	1.5E+02	1.2E+03

*Corrected parameters were calculated as follows in our sample: RMMSDc = RMSSD/meanRR^3.7^, pNN50c = pNN50/meanRR^5.3^, SDNNc = SDNN/meanRR^2.8^, FFT TPc = TP/meanRR^6.3^, FFT HFc = HF/meanRR^6.3^, FFT LFc = LF/meanRR^5.0^, FFT LF/HFc = LF/HF x meanRR^1.8^.*

Supplementary Figures S1, S2 present the Spearman correlations between HRV parameters and HR. Significant correlations found between the standard HRV parameters and HR (r ranging from −0.802 to 0.250; all *p* ≤ 0.009) disappeared after the correction procedure (r ranging from −0.120 to 0.033; *p* ≥ 0.220).

Table 3 shows associations between BC measures and HRV parameters. Concerning to time-domain HRV parameters, the model 1 (basic confounders tested were sex, PHV offset, parent education university level and wave but none were included after performing stepwise analyses) showed negative associations of BMI with RMSSD and pNN50 (β = −0.207 and β = −0.240, respectively; both *P* < 0.032). Also, WC/height ratio was negatively associated with pNN50 (β = −0.194, *p* = 0.046). FMI and BFP from DXA were negatively associated with RMSSD, pNN50 and SDNN (β ranging from −0.203 to −0.272; *p* ranging from 0.005 to 0.036). In regard to HRV parameters in frequency-domain, BMI and FMI were negatively associated with HF (β = −0.214 and β = −0.208, respectively; both *P* < 0.032). Also, BMI, WC, FMI, and FFMI were positively associated with LF/HF (β ranging from 0.201 to 0.283; both *p* ranging from 0.002 to 0.038). In model 2 (same confounders as model 1 plus mean HR) and model 3 (selected basic confounders by the stepwise models in model 1, and corrected HRV parameters as outcomes), the previously found associations between BC measures and mainly HRV indicators of PA (RMSSD, pNN50, and HF) in time- and frequency-domain were no longer significant (all *p* > 0.05).

**TABLE 3:** Standardized beta coefficients from linear regression models on the associations of body composition measures with standard and corrected HRV parameters.

	**RMSSD**	**pNN50**	**SDNN**	**FFT TP**	**FFT HF**	**FFT LF**	**FFT LF/HF**
**Model 1: Standard HRV parameters; Confounders^†^: none**							
BMI (kg/m^2^)	−**0.207**	−**0.240**	–0.158	–0.117	−**0.214**	–0.018	0.283^*^
WC (cm)	–0.120	–0.148	–0.052	–0.042	–0.125	0.024	**0.201**
WC/Height	–0.152	−**0.194**	–0.076	–0.065	–0.146	–0.031	0.159
DXA FMI (kg/m^2^)	−**0.232**	−0.272^*^	−**0.203**	–0.138	−**0.208**	–0.042	0.250^*^
DXA BFP (%)	−**0.221**	−0.261^*^	−**0.220**	–0.148	–0.168	–0.084	0.146
DXA FFMI (kg/m^2^)	–0.062	–0.067	–0.005	–0.019	–0.122	0.048	**0.225**
DXA Total VAT (g)	–0.048	–0.090	–0.019	–0.005	–0.054	0.069	0.121
DXA VAT FMI (g)	–0.090	–0.134	–0.062	–0.036	–0.095	0.019	0.082
**Model 2: Standard HRV parameters; Confounders^†^: HR**							
BMI (kg/m^2^)	–0.034	–0.058	0.003	0.046	–0.052	0.131	**0.242**
WC (cm)	–0.003	–0.026	0.057	0.067	–0.015	0.122	0.168
WC/Height	0.095	0.063	0.158	0.169	0.086	**0.179**	0.094
DXA FMI (kg/m^2^)	–0.028	–0.059	–0.015	0.055	–0.015	0.135	**0.201**
DXA BFP (%)	–0.008	–0.039	–0.026	0.051	0.035	0.097	0.086
DXA FFMI (kg/m^2^)	–0.039	–0.042	0.017	0.003	–0.099	0.068	**0.218**
DXA Total VAT (g)	0.050	0.013	0.075	0.100	0.043	0.132	0.092
DXA VAT FMI (g)	0.111	0.074	0.131	0.159	0.103	0.159	0.023
**Model 3: Corrected HRV parameters; Confounders^†^: years PHV offset**							
BMI (kg/m^2^)	0.026	–0.042	0.010	0.109	–0.014	0.140	**0.198**
WC (cm)	0.107	–0.006	0.101	0.156	0.065	0.129	0.112
WC/Height	0.108	0.004	0.114	0.179	0.069	0.168	0.114
DXA FMI (kg/m^2^)	0.012	–0.058	–0.018	0.117	0.016	0.136	0.163
DXA BFP (%)	0.012	–0.055	–0.036	0.105	0.058	0.085	0.061
DXA FFMI (kg/m^2^)	0.024	–0.011	0.040	0.033	–0.073	0.090	0.183
DXA Total VAT (g)	0.102	–0.020	0.062	0.121	0.058	0.117	0.089
DXA VAT FMI (g)	0.157	0.057	0.109	0.181	0.110	0.110	0.020

BMI, body mass index; WC, waist circumference; DXA, dual energy X-ray absorptiometry; FMI, fat mass index; BFP, body fat percentage, FFMI, fat-free mass index; VAT, visceral adipose tissue; PHV, peak height velocity. See full name of all the heart rate variability in time and frequency-domain (units in ms and ms^2^, respectively) parameters in Table 1. ^†^Confounders on the model were selected by stepwise regression analyses (i.e., all basic confounders considered in the three models were sex, PHV offset, parental education and wave). Additionally, mean HR was introduced as a confounder in the model 2. Stepwise analyses for confounders did not introduced any of the confounders tested in model 1, only mean HR in model 2 and PHV offset in model 3. Bold numbers indicate *p* < 0.05. ^*^Indicates *p* < 0.01.

**FIGURE 1:**
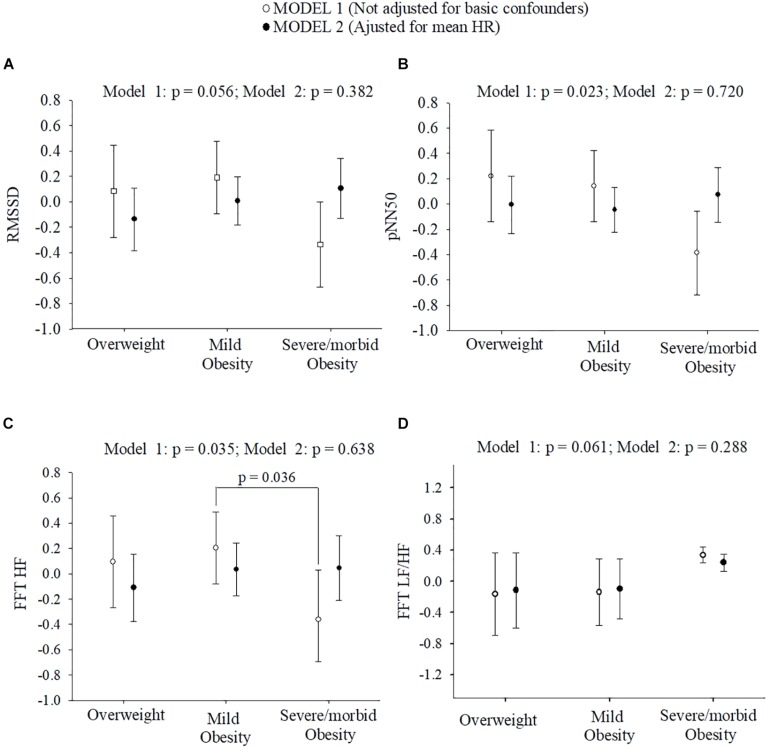
Normal scores of HRV parameters in time and frequency-domain across children with overweight/obesity. **(A,B)** show differences of HRV parameters in time-domain across children with overweight/obesity. **(C,D)** show differences of HRV parameters in frequency-domain across children with overweight/obesity. Adjusted means and 95% confidence intervals are presented. Bonferroni *post hoc* analysis did not show significant differences for pNN50 across children with different weight status. See full name of all the heart rate variability parameters in Table 1.

Figure 1 depicts differences on standard HRV parameters across weight status groups in model 1 and model 2. Concerning HRV parameters in time-domain, there were (model 1) no significant differences for RMSSD (*p* = 0.056) and SDNN (*p* = 0.299), but significant differences across weight status for pNN50 (*p* = 0.023). Bonferroni *post hoc* analysis did not show significant differences for pNN50 across weight status groups.

Regarding frequency-domain HRV parameters, no significant differences were reported for LF (*p* = 0.557) and TP (*p* = 0.380) across weight status groups. Importantly, children with severe/morbid OB showed significantly lower values on HF in respect to peers with mild OB (*p* = 0.036) and borderline differences in LF/HF across weight status (*p* = 0.061). All these differences disappeared after additional adjustment for mean HR (model 2, all *p* > 0.05).

## Discussion

### Main Findings

The main findings of this study were: BC measures (BMI, WC/Height, FMI, and BFP) were negatively associated with HRV indicators of PA (RMSSD, pNN50, and HF) and children with severe/morbid OB had lower values of HRV (RMSSD, pNN50, and HF) compared to their peers with OW and mild OB. However, nearly all associations of BC measures with HRV parameters, as well as differences across weight status groups, were fully explained by HR.

### Associations Between BC Measures and HRV Parameters

Some HRV parameters in time and frequency-domain are considered indicators of PA (i.e., RMSSD, pNN50, and HF). For time-domain parameters, we found inverse associations of BMI with RMSSD and pNN50. Also, FMI and BFP were negatively associated with RMSSD, pNN50 and SDNN. Accordingly, Gutin et al. (2000) found negative significant associations of fat mass and fat-free mass (from DXA) with RMSSD. Likewise, we did not find associations between VAT and HRV in our sample, in accordance with this previous study (Gutin et al., 2000). In contrast to Gutin et al. (2000), we did not find associations between FFMI and RMSSD. HRV methodological differences, such as different instrument (i.e., ECG vs. HR monitor), differences in sample characteristics (i.e., children with obesity vs. children with overweight/obesity in our study) and different confounders could be responsible for these inconsistent findings between studies.

Furthermore, in relation to HRV parameters in frequency-domain we found negative associations of BMI, and FMI, with HF used as indicator of PA. Also, we found positive associations of BMI, WC, FMI, and FFMI with LF/HF. In the same line, previous studies found similar results in children with OW/OB (Baum et al., 2013; Santos-Magalhaes et al., 2015). For example, Baum et al. (2013) found negative and positive associations of BMI with HF and LF/HF ratio, respectively. Santos-Magalhaes et al. (2015), similar to our study, showed significant positive associations between WC, as indicator of central fat, and LF/HF ratio. Some authors have considered LF/HF ratio as an indicator of sympatho-vagal balance (Pagani et al., 1986; Malliani et al., 1991). Under this assumption, our findings suggest that higher BMI, WC and fat mass levels are associated with higher predominance of sympathetic activity over PA in children with overweight/obesity. However, physiological significance of LF/HF ratio is not clear in the literature (Goldstein et al., 2011; Billman, 2013). For example, has been reported that beta-adrenergic receptor blockade combined with parasympathetic denervation supposed an increase of LF/HF ratio from 1.1 to 8.4, which suggest a “false” sympathetic dominance (Randall et al., 1991; Billman, 2013). Importantly, some interventions such as myocardial ischemia or exercise did not increase LF, instead provoked a significant reduction in LF power (Houle and Billman, 1999). The interpretation of the LF/HF ratio findings as indicator of sympatho-vagal balance should be considered with caution [see Billaman article for revision (Billman, 2013)].

### HRV Differences Between Weight Status Groups

Children with severe/morbid OB presented lower RMSSD, pNN50 and HF (indicators of PA) and higher LF/HF ratio compared to their peers with OW and mild OB in our study. These differences were borderline significant for RMSSD and LF/HF ratio, which can be assumed as lack of statistical power due to our relatively small simple size (*N* = 107). Vanderlei et al. (2010) found significantly lower RMSSD, pNN50, and HF values in children with OB compared to NW children. Likewise, Birch et al. (2012) found lower HF values in children with OW/OB compared to their peers with NW. Accordingly, Rodríguez-Colón et al. (2011) reported lower values in SDNN and LF in children with OB compared to children with NW and OW. Also, in that study, higher LF/HF ratio and HR values were reported in children with OB compared with their NW and OW peers. Otherwise, Redón et al. (2016) showed similar SDNN values across children with OW, mild and severe OB, which concurs with present findings. These differences might be explained by different instruments and durations for the HRV assessment (Task Force, 1996), i.e., 9 h of continuous ECG in the Rodríguez-Colón et al. (2011) study, 15 min with ECG in the Redón et al. (2016) study, and 10 min with HR monitor in our study. Also, the differences in sample size and characteristics between studies should be considered. For example in our study and the study of Redón et al. (2016) the sample size was relatively low (*n* = 107 and 64, respectively) and in general we did not find significant differences on HRV parameters between weight status groups (only significant for HF in our study). However, the sample size on Rodríguez-Colón et al. (2011) study was higher (*n* = 616) and they found lower values in SDNN.

### The Dependence of HRV on HR and Health Outcomes

This study provides values of HRV_c_ in children with OW/OB with the values published previously in healthy weight children (Ga̧sior et al., 2018). The values of HRV_c_ in healthy weight children were higher than in children with OW/OB, further supporting the notion of obesity associated with a worse HRV profile. Future research should test whether differences in HRV_c_ observed between healthy weight children and our sample of children with OW/OB are clinically relevant. On the other hand, some methodological differences between the study of Ga̧sior et al. (2018) and our study should be considered to compare these values: sample size was higher than ours (*n* = 312 and *n* = 107, respectively) compromising the representativity of the results, the instrument to record HRV signal (i.e., ECG vs. HR monitor), the range of age (i.e., 6–13 and 8–11 years, respectively) and, the HF band was fixed at different frequencies (0.5 Hz vs. 0.4 Hz, respectively).

The majority of the previous studies did not consider the dependence of HRV on HR in children with OW/OB (Rodríguez-Colón et al., 2011; Baum et al., 2013; Da Silva et al., 2014; Redón et al., 2016). It is important to note that in our study nearly every association between BC measures and HRV parameters disappeared after considering mean HR, either as confounder or after calculating the HRV_c_ proposed by Sacha et al. (2013). Similarly, differences across weight status groups also disappeared after considering the influence of mean HR. Our study suggests that HRV differences across weight status groups are explained by differences in mean HR, independently of the method used to remove the HRV dependence on HR. It should be underlined that standard HRV actually provides information on two quantities, i.e., on HR and its variability and it is hard to determine which of these two plays a principal role in the clinical value of HRV (Sacha, 2013, 2014b). Furthermore, the association between HRV and HR is not only a physiological phenomenon but also a mathematical one, which is due to non-linear (mathematical) relationship between RR interval and HR (Sacha and Pluta, 2008). However, by removing HRV dependence on HR one explores the HR contribution to the physiological and clinical significance of HRV in a given clinical context (Sacha, 2013, 2014b).

To our knowledge, the influence of HR on the associations between BC and HRV parameters has not been previously tested in children. However, the influence of HR on the associations between other health outcomes and HRV have been reported in children with NW and young adults (Grant et al., 2013; Van De Wielle and Michels, 2017). In the context of physical fitness and health, associations between HRV and VO_2_max were lost after removing the dependence of HRV on HR, so these associations are explained by the relationships between mean HR and VO_2_max (Grant et al., 2013). Also, the contribution of HR to the clinical value of HRV has been reported (Sacha, 2014a,c). In fact, Sacha et al. (2013) showed that prediction capacity of HRV in relation to cardiovascular mortality worsened after removing the dependence of HRV on HR, especially for populations and events where HR was a strong risk factor (Sacha, 2014c). Similar to previous findings, our study suggests that the relationships between health parameters (i.e., VO2max, blood biomarkers, BC measures) and HRV should be considered the influence of HR. This is an important observation that should be tested in future studies with different health outcomes in children with OW/OB and other populations (i.e., older adults, older adults with severe/morbid obesity, older adults with cardiovascular diseases).

### Strengths and Limitations

Several limitations need to be acknowledged in our study: (1) the cross-sectional design of this study does not allow for causal interpretation; (2) we did not use a gold standard for the HRV measurement, however, the RS800CX has demonstrated to be valid and reliable for its assessment; (3) some studies have found HRV parameters to be affected by the breathing (Wessel et al., 2009; Sidorenko et al., 2016) although it depends on the HRV parameter analyzed (Hill and Siebenbrock, 2009), so we decided to not disturb the resting status of participants and to let them breath naturally; (4) we do not include children with NW in our sample to compare HRV differences with children with OW, mild, severe and morbid OB; (5) The relatively small sample size in different subgroups based on BMI. The strengths of our study were: (1) BC was measured with the method gold-standard (DXA); (2) we tested the influence of potential confounding variables such as sex, PHV offset, socioeconomic status measured as parental education and wave; and (3) to the best of our knowledge this is the first study investigating the influence of HR on the associations between BC measures and HRV in children with OW/OB, which has demonstrated to completely affect the findings.

## Conclusion

Our study suggests that BC measures are negatively associated with HRV parameters, indicators of PA, but the associations found seem to be explained by mean HR. Likewise, children with severe/morbid OB had lower HRV values than their OW/mild OB peers, which could be explained by the higher HR values in children with severe/morbid OB compare to peers with OW/mild OB. The “simplest” concept of HR seems to be explaining the associations between BC measures and the “complex” concept of HRV in children with OW/OB. Likewise, the HR assessment and interpretation are easier than HRV parameters. Thus, future research should investigate the dependence of HRV on HR before concluding associations or effects on HRV parameters. Clinicians should take into consideration the assessment of HR and not only the measurement of HRV alone in children with weight disturbances.

## Data Availability

All datasets generated for this study are included in the manuscript and/or the Supplementary Files.

## Ethics Statement

This study was conducted according to the Declaration of Helsinki. The protocol was approved by the Committee for Research Involving Human Subjects at the University of Granada (Reference: 848, February 2014). All parents had received information about the study and gave written informed consent in accordance with the Declaration of Helsinki.

## Author Contributions

JM, JM-G, CC-S, and FO made substantial contributions to design the experiment and study design. AP-F, JM, JM-G, PM-G, MR-A, CC-S, IE-C, JS, and FO made substantial contributions to acquisition, analysis, or interpretation of data for the manuscript. All authors drafted and revised the manuscript critically for important intellectual content. All authors approved the final version of the manuscript.

## Conflict of Interest Statement

The authors declare that the research was conducted in the absence of any commercial or financial relationships that could be construed as a potential conflict of interest.

## Footnotes

1 http://profith.ugr.es/activebrains?lang=en (Clinical Trial: NCT02295072).
