# Supplementary material for: The Role of Heart Rate on the Associations Between Body Composition and Heart Rate Variability in Children With Overweight/Obesity: The ActiveBrains Project
Source: Front Physiol. 2019 Jul 16;10:895. doi: 10.3389/fphys.2019.00895 (PMC6646801; doi:10.3389/fphys.2019.00895)
Supplement: Supplementary file 1 [file Data_Sheet_1.docx]

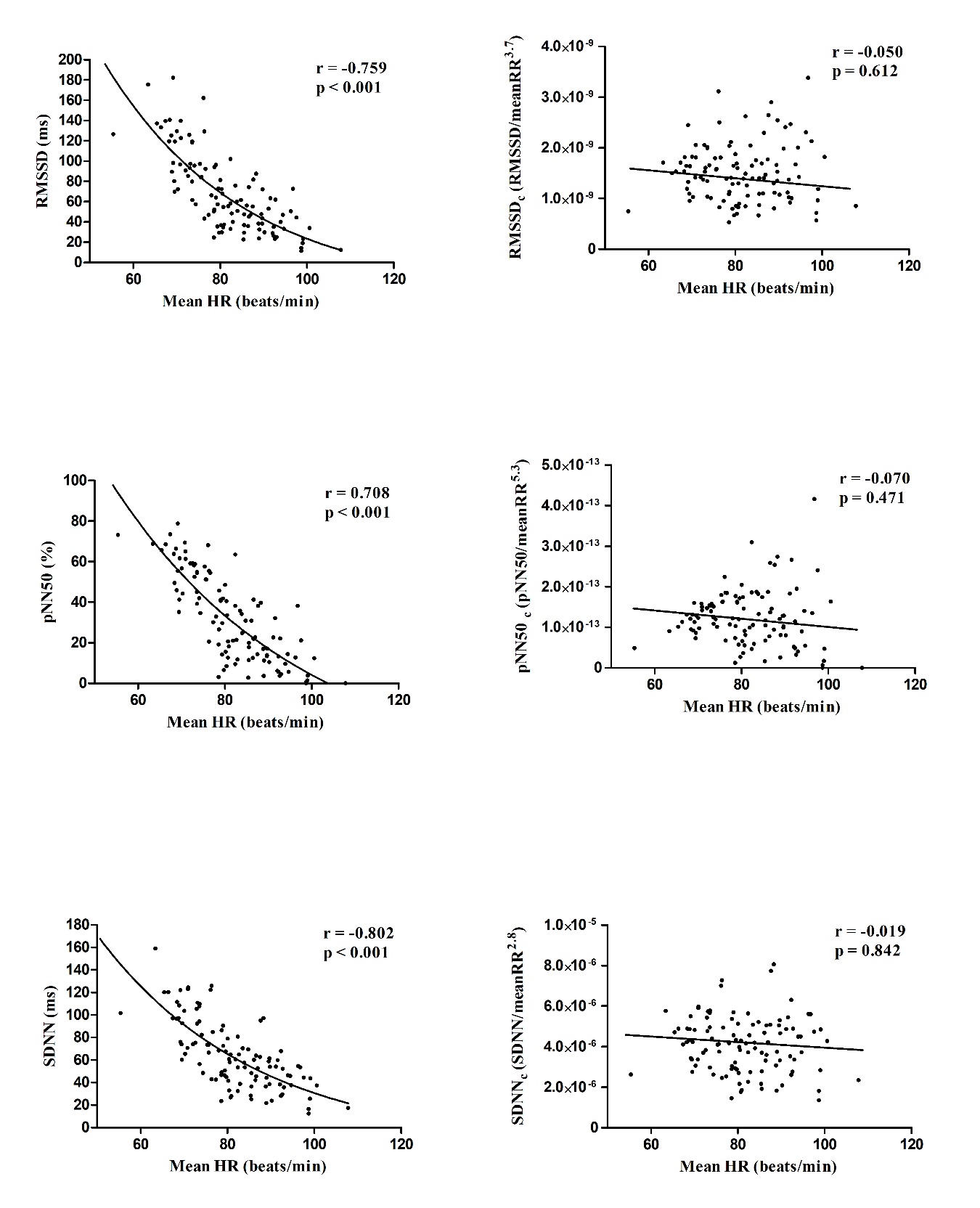
**Figure S1.** Scatter plots showing the Spearman correlations of time domain standard (left panels) and corrected (right panels) HRV parameters with mean heart rate.


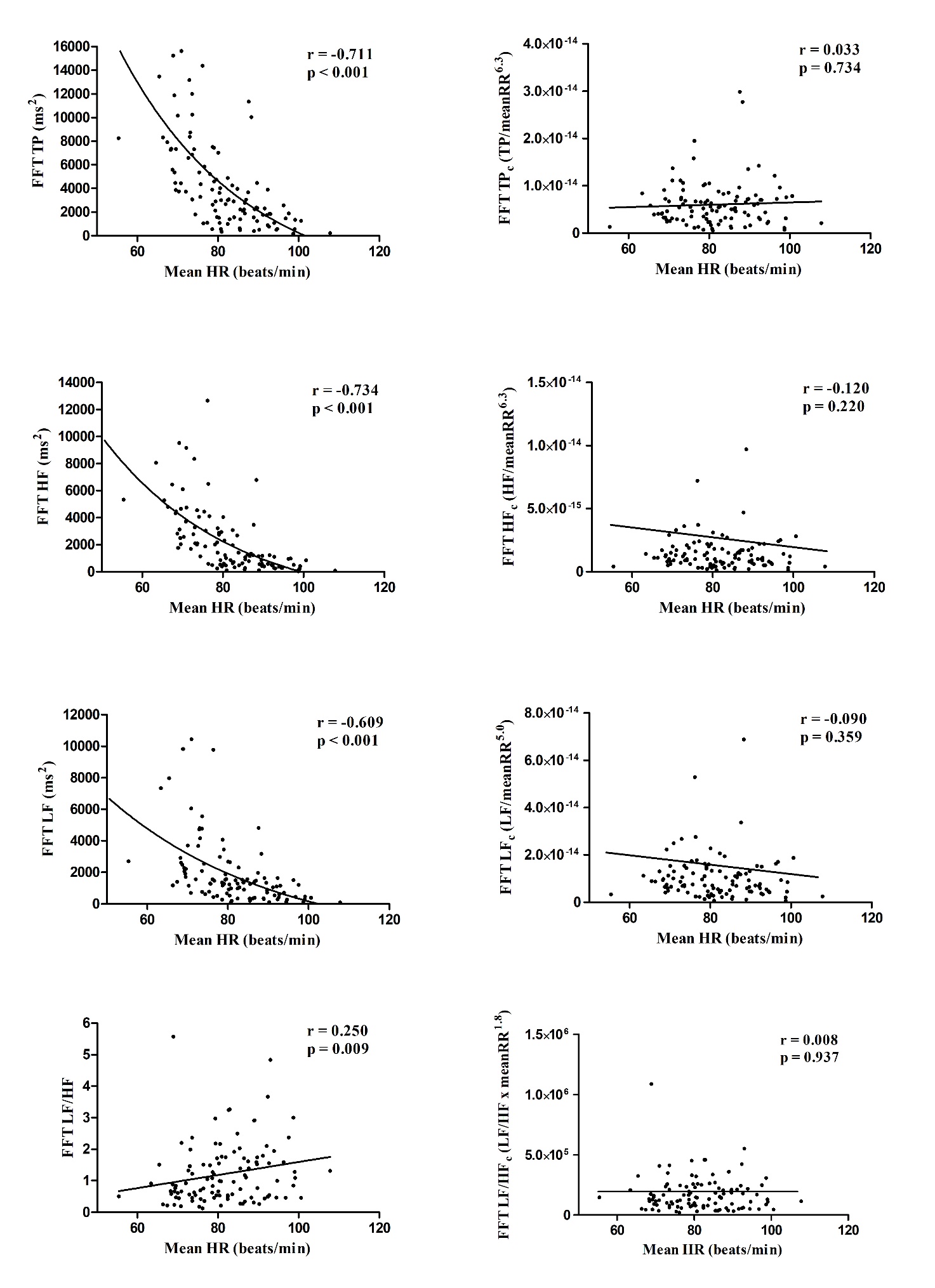


**Figure S2.** Scatter plots showing the Spearman correlations of frequency domain standard (left panels) and corrected (right panels) HRV parameters with mean heart rate
